# Identifying risks in dementia care: Insights from a qualitative study with clinical pharmacists

**DOI:** 10.1371/journal.pone.0325472

**Published:** 2025-06-24

**Authors:** Alice Burnand, Kumud Kantilal, Abi Woodward, Cini Bhanu, Jill Manthorpe, Mine Orlu, Greta Rait, Kritika Samsi, Liz Sampson, Victoria Vickerstaff, Jane Wilcock, Yogini Jani, Nathan Davies

**Affiliations:** 1 Research Department of Primary Care and Population Health, Centre for Ageing Population Studies, University College London, London, United Kingdom; 2 Centre for Psychiatry and Mental Health, Wolfson Institute of Population Health, QMUL, London, United Kingdom; 3 NIHR Policy Research Unit in Health and Social Care Workforce, King’s College London, London, United Kingdom; 4 NIHR Applied Research Collaborative (ARC) South London, King’s College London, London, United Kingdom; 5 Research Department of Primary Care and Population Health, PRIMENT Clinical Trials Unit, University College London, London, United Kingdom; 6 Centre for Evaluation and Methods, Wolfson Institute of Population Health, QMUL, London, United Kingdom; 7 Research Department of Practice and Policy, School of Pharmacy, University College London, London, United Kingdom; 8 Centre for Medicines Optimisation Research and Education, University College London Hospitals NHS Foundation, London, United Kingdom; 9 Research Department of Pharmaceutics, UCL School of Pharmacy, University College London, London, United Kingdom; University of Minnesota, UNITED STATES OF AMERICA

## Abstract

**Introduction:**

People with dementia experience complex healthcare needs. Clinical pharmacists play a crucial role in optimising medication management and ensuring patient safety within the primary care setting. However, little is known about the specific barriers and challenges they face when delivering dementia care. This study investigates the safety of dementia care in the community, focusing on the experiences of clinical pharmacists.

**Methods:**

A qualitative study using semi-structured interviews with clinical pharmacists. Data analysis employed codebook thematic analysis, guided by the SEIPS 2.0 framework.

**Results:**

Thirteen clinical pharmacists were interviewed. Key risks include variations in pharmacist expertise, communication barriers, limited resources, and systemic challenges. Using the SEIPS 2.0 framework, the complex interactions between work systems (people, tools, tasks, organisation and environment) and work processes were identified, which impact safety outcomes for both clinical pharmacists and people with dementia.

**Conclusions:**

Interviews revealed a spectrum of risks associated with dementia care in primary care. This study highlights the complex interplay of factors influencing the safety of dementia care. A proactive, multifaceted approach addressing training, interprofessional collaboration, and system-level adaptations is crucial to mitigate these risks and enhance patient safety in dementia care.

## Introduction

Increasing life expectancy globally together with increasing prevalence of multiple long-term conditions, such as dementia, are placing significant strain on primary care systems [1,2]. Many factors, including cognitive decline, comorbidities, presence of polypharmacy and drug side effects, contribute to high and multidimensional levels of need among many people with dementia [3–5].

Effective dementia care requires a holistic approach that addresses the complex physical, psychological, and social needs of people with dementia [6,7]. Cognitive decline and multiple comorbidities can lead to increased risks of harm for people with dementia, such as falls [8,9] and inappropriate prescribing [10]. Memory loss and language difficulties may give rise to challenges in communicating medication use and side-effects [11–13]. The evidence base to guide prescribing for this population is limited, as people with dementia are often excluded from clinical trials and not always considered in guidelines [14]. Polypharmacy is also common in older people with dementia and can lead to a range of adverse outcomes [15,16].

Evidence-based structured medication reviews aim to reduce problematic polypharmacy and inappropriate prescribing [17]. Clinical pharmacists are highly qualified experts in medicines management and a large part of their role involves completing medication reviews [17]. In recent years there has been expansion of the clinical pharmacist role in complex disease management as part of the multi-disciplinary team (MDT) in primary care [18,19]. Current policy within the NHS in England is to expand the clinical pharmacy workforce within MDTs in general practice [20]. Evidence has shown their involvement improves quality of care for patients and can reduce medication-related errors [21,22].

Despite evidence of MDT involvement in improving health outcomes [23], health care professionals, including clinical pharmacists, experience multidimensional challenges when providing dementia care. This can include poor organisational support, stress and lack of capability [24,25]. Despite the potential for clinical pharmacists to improve patient outcomes through medication management [26,27], research on their role in dementia care regarding safety and risk management is limited [28]. By understanding the challenges associated with supporting dementia care, healthcare systems can become better equipped to proactively address risks of harm [29]. This can reduce potential harm to people with dementia and improve the delivery of services by clinical pharmacists [29–31].

In this paper, we have adopted a patient safety approach that incorporates human factors, shifting the focus from individual staff knowledge and skills to the “interactions between people and their environment that contribute to performance, safety, and health” [32]. This perspective is valuable as it accounts for the broader systems that influence both staff and patient outcomes. We use The System Engineering Initiative for Patient Safety (SEIPS) 2.0 as a framework to gain insight into structures, processes and outcomes in healthcare [33]. By examining these relationships and outcomes, the framework can help identify areas for improvement in safety during the delivery of care. SEIPS 2.0 can be used as a guide and problem-solving tool to learn from patient safety incidents, to prevent them reoccurring. The framework places patients and staff at the centre of work systems and helps understand problems with the tasks they do, the tools and technologies they need to provide care and the environments that impact on care delivery. The work systems produce, support or inhibit the work processes which take place, for example how consultations are delivered.

The SEIPS 2.0 framework helps understand work processes of care (performed by patients, carers, health care professionals, or in collaboration) that could contribute to poor care or incidents, as well as including what works well. These processes in turn shape work outcomes.

Whilst research into dementia care and clinical pharmacy utilising the SEIPS 2.0 framework is limited, it is well-established for improving health and healthcare processes and outcomes and has been utilised in analysing care needs for older adults [34] and chronic health conditions such as diabetes [35,36].

This study aimed to understand the system level safety for people with dementia and for clinical pharmacists, by exploring risks as identified by clinical pharmacists, when supporting dementia care in the community.

## Materials and methods

### Research design

A qualitative study, using semi-structured interviews with clinical pharmacists. Interviews were analysed using codebook thematic analysis [37] underpinned by the application of the SEIPS 2.0 framework [33].

### Ethical approval

Approval was obtained from the Health Research Authority (HRA) and Health and Care Research Wales (23/LO/0054) and University College London Research Ethics Committee (3344/006).

### The SEIPS 2.0 framework

**Fig 1:**
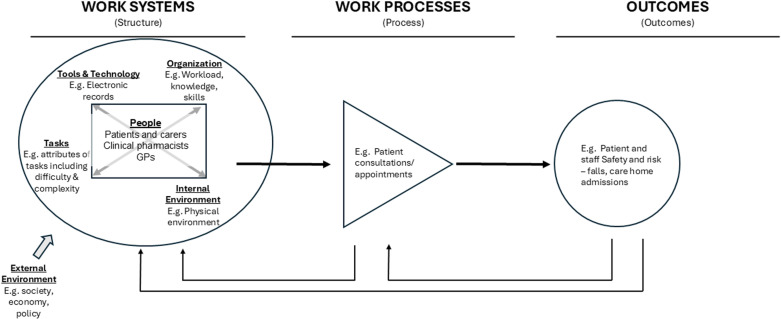
SEIPS 2.0 framework adapted from Holden et al., 2014 [33].

The SEIPS 2.0 framework comprises three primary components: the work system, work processes, and outcomes [33]. We utilised this framework to understand how various factors within the primary care system contribute to risks for both clinical pharmacists and patients (see Fig 1). In our research, the outcomes we focussed on were safety and risk of harm to patients with dementia and risk and safety for clinical pharmacists when delivering dementia care within the context of England’s primary care services.

### Recruitment and sample

Participants were recruited using purposive sampling with an aim to recruit a diverse sample across age, sex, ethnicity, and location of work across England. Our sample size (n = 13) was guided by the principles of information power, which considers the study aim, sample specificity, quality of dialogue and analysis strategy [38]. Considering these factors, we estimated recruiting approximately 10 clinical pharmacists due to our narrow aim and expected high quality data with rich content. Participants were eligible if they were a clinical pharmacist working in primary care in England. Participants were recruited through GP/primary care practices, National Health Service (NHS) networks, known contacts of the research team, and supplemented by snowballing methods. A national survey was conducted by the research team in 2022−23 and participants who completed this survey and expressed interest in further involvement were invited to participate in interviews. Flyers and posters were also distributed on social media, mailing lists and distributed to clinical teams. Participants who were interested in the study were encouraged to contact the research team. Recruitment started on 01/07/2022 and finished on 01/03/2023.

### Data collection

Interviews were guided by a semi-structured interview schedule developed collaboratively with our Patient and Public Involvement and Engagement (PPIE) panel, drawing upon the findings of the initial survey and a systematic review [30]. Our PPIE panel is representative of two family carers with lived experience of supporting someone with dementia whilst also receiving support from a clinical pharmacist. The interview schedule was piloted and refined prior to conducting interviews.

Written informed consent was obtained from participants prior to interviews and demographic information, including age, ethnicity, level of education, was collected from participants. Between December 2022 and March 2023 interviews were conducted by either telephone or video call (using Microsoft Teams or Zoom) by a member of the research team (AW) and were audio recorded. All interviews lasted between 30 and 60 minutes and were transcribed verbatim. Another researcher (AB) verified the accuracy of the transcripts by comparing them to the audio recordings. All participants were offered a £20 gift voucher for their time.

### Data analysis

Transcripts were uploaded to NVivo 14 [39] and analysed using codebook thematic analysis taking a mixture of an inductive and deductive approach [37] using the SEIPS 2.0 framework [33]. We initially developed our coding frame using a deductive approach based on the SEIPS 2.0 framework we then then iteratively refined the coding frame inductively based on the interview participants responses.

Two researchers (AB and KK), read and independently coded all transcripts, meeting regularly with YJ and ND to discuss. All codes were discussed with the wider team who provided feedback. The research team is multidisciplinary with experts within primary care, social care, sociology and epidemiology, all of whom had experience in qualitative research and thematic analysis. The research team was of mixed gender, age and ethnicity.

## Results

### Sample characteristics

Thirteen clinical pharmacists were interviewed. Of these, ten completed the demographic questionnaires (See Table 1). We did not receive responses from the remaining three participants.

**Table 1:** Demographic characteristics of participants (n = 10).

		Clinical Pharmacist
Age (years in bands)	18–35	3
	36–45	1
	46–55	4
	56–65	1
	Not answered	1
Gender	Female	7
	Male	3
Ethnic group	White	5
	Any other White background	1
	Asian or Asian British	3
	Other ethnic group	1
Education	Undergraduate Degree	4
	Additional Master’s Degree	5
	Postgraduate Diploma	1
Professional Grade	NHS Band 6 (junior pharmacist)	4
	NHS Band 7 (senior pharmacist)	1
	NHS Band 8 (senior pharmacist team leader)	5
Years working as clinical pharmacist	Less than 1 year	1
	2–3 years	1
	More than 5 years	8
Years experience in primary care	Less than 1 year	1
	2–3 years	3
	4–5 years	1
	More than 5 years	5
Work location	London	3
	Southwest England	1
	Northeast England	2
	Southeast England	1
	Northwest England	3

**Fig 2:**
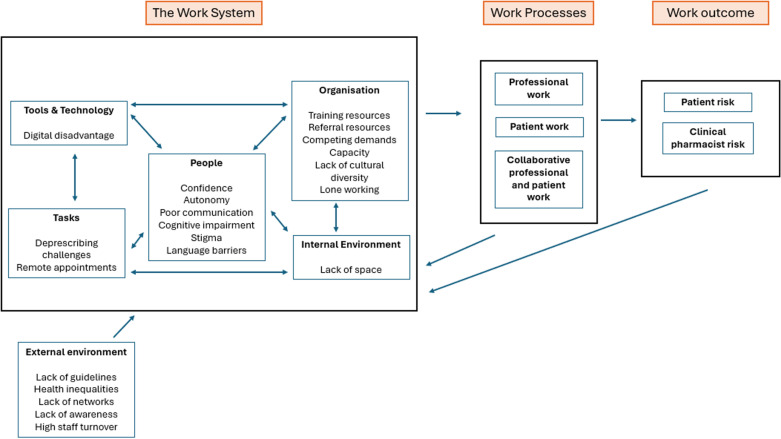
Risks identified by clinical pharmacists when interviewed presented using the SEIPS 2.0 framework. Work systems are an interconnected set of factors, which shape and produce the work processes. These work processes are depicted as patient, professional and the collaborative work between the two groups. The work outcome represents the risks for patients and clinical pharmacists in dementia care. All systems interact with each other and influence the risk occurring. The person is placed at the centre of the work system to highlight how the design should support individuals, and how pharmacists’ performance is affected by interactions within the other work systems. The feedback loops indicate how changes in work systems can occur due to the work outcome [33].

The interviews highlighted a spectrum of risks associated with providing dementia care in primary care settings. By mapping these themes onto the SEIPS 2.0 framework, we identified the interplay and challenges within the work systems components, and the work processes that can impact the outcomes. The outcomes constitute the safety and risks for both clinical pharmacists and patients involved in dementia care. Fig 2 illustrates our findings within the SEIPS 2.0 framework, showing the interconnected factors in work systems and work processes that lead to overall risk. The specific risks identified in the transcripts have been included in this visualisation. Table 2 also presents the perceived risks identified in the transcripts that relate to both work systems and processes in the SEIPS 2.0 framework.

**Table 2:** Work systems, processes and outcomes identified within the SEIPS 2.0 framework, and the perceived risk in providing dementia care.

WORK SYSTEMS	WORK PROCESSES Professional Work, Patient Work, Collaborative Work		
	Challenges	Explanation related to work processes	Perceived outcome
Tools and Technology	**Use of digital technology**	While many patients and their family members are confident in using the internet, some patients, such as many of those with cognitive impairment and older people, are disadvantaged by the increase in use of digital technology as they may struggle to access and utilise the internet.	While this is beneficial for many, some patients are not able to access useful resources to support their care, contributing to health inequalities.
Tasks	**Challenges with deprescribing when a GP does not agree.**	Clinical pharmacists are unable to complete medication reviews and subsequent deprescribing.	Risk of patient polypharmacy or remaining on anticholinergic drugs that worsen their dementia symptoms. Lack of trust between patient/carer and primary care team following perceptions of conflict/disagreement
	**Challenge with deprescribing if a patient wants to remain taking certain medications.**		
	**Increasing use of remote appointments.**	Challenges at times with effectively assessing patients over the phone during remote appointments.	While potentially beneficial in terms of reduced travel time these can lead to suboptimal care for the patient.
People	**Confidence as a clinical pharmacist**	Variations in confidence and ability to support people with dementia, such as with communication with the patient.	Can lead to suboptimal care for the patient and avoidance of working with this patient group.
	**Autonomy variations**	Variation in autonomy for pharmacists and what they specialise in beyond CPPE.	This may negatively impact their professional development and the range of patients they can support.
	**Poor communication**	Challenges for clinical pharmacists who do not have a supportive primary care team to help them undertake their duties effectively.	Can lead to suboptimal care for the patient.
	**Presence of cognitive impairment**	Challenges with communication between clinical pharmacists and those patients with cognitive impairment. Patients’ cognitive ability impacts medication adherence	Can lead to suboptimal care for the patient. Risks of overdosing or missing medication if no carer/care worker support in place.
	**Stigma**	Stigma in minority ethnic cultures about mental illness including dementia, and acceptance of a diagnosis and support from primary care teams, including clinical pharmacists, for such long-term conditions.	May prevent patients from seeking support or starting medication, and limit carers’ support.
	**Language barriers**	Language barriers between health and care professional and those for whom English is not their first language.	Can lead to suboptimal care for the patient and carers if interpreter services are not available.
Organisation	**Differences in training resources depending on location.**	Leads to variation in patient care and a geographical “post-code lottery”.	Some patients are not able to access useful resources to support with their care due to their location, contributing to health inequalities.
	**Differences in referral resources depending on location**		
	**Competing demands over support with other long-term conditions.**	Dementia is sometimes overlooked compared to other long-term conditions and is not prioritised in reviews by clinical pharmacists.	Dementia symptoms are not prioritised during a review of the patient and may lead to suboptimal care for the patient.
	**Capacity pressures**	Clinical pharmacists being less able to see patients face to face, either at home or in practice. Not enough capacity for longer appointments and follow-ups.	Limitations can result in follow-ups not happening and suboptimal care.
	**Resources mainly tailored for Western cultures.**	Resources used by clinical pharmacists and other healthcare professionals are not culturally inclusive.	Some patients are not able to utilise useful resources to support with their care, contributing to health inequalities.
	**Lone working**	Safety concerns for clinical pharmacists completing home visits alone and meeting patients who may be distressed or have behavioural challenges.	Clinical pharmacists may be at risk of harm and not be able to provide care to patient.
Internal Environment	**Lack of space in GP/primary care practice to see patients**	Leading to pharmacists working remotely.	Do not get the benefits of seeing patients or carers face-to-face.
External Environment	**Lack of guidelines or protocol or awareness of these**	Clinical pharmacists lack of guidance when supporting patient groups.	Lack of knowledge of effective approaches to support people with dementia.
	**Health inequalities**	Health inequalities impacting patient care, such as medication adherence and in some cases affordability of prescriptions.	May prevent patients from starting/continuing medication.
	**No professional networks**	Lack of ability to learn from others and contribute to professional development as a clinical pharmacist.	May impact their professional development.
	**Lack of awareness**	Patients unaware of the clinical pharmacy services and their roles.	The service is not utilised or optimised to support patients.
	**High turnover of carehome and home care staff**	Inconsistent information for care home residents or those with cognitive impairment who rely on external support.	Can impact decision-making and lead to suboptimal care for the patient.

The work system, comprising tasks, tools and technology, people and organisation influenced the professional work, patient work and collaborative processes, which, depending on internal and external environmental factors, led to potential risks for patients as well as the clinical pharmacists as detailed in the following sections:

### People

In the SEIPS 2.0 framework, “people” describes the individual characteristics of both patients, healthcare professionals and carers, as well as collective characteristics such as team dynamics which can influence care processes and outcomes.

Collaboration within primary care teams varied among participants, posing significant challenges to how effectively clinical pharmacists could deliver their services. This lead to inconsistent care quality especially for complex medical decisions without sufficient clinical support and could lead to delays in prescribing or inappropriate prescribing. Some pharmacists noted that strong cohesion and support within a team help facilitate complex and challenging cases:

*“If you’ve got a supportive GP, so if you have a supportive team that will really go a long way, …you can always just ask them for help, if I ever felt out of my depth.” [CP04].*

However, the level of expertise in dementia varied among pharmacists due to differences in individual professional development and chosen areas of specialisation, which influenced their confidence in supporting people with dementia. Some pharmacists had proactively sought and opted for additional training beyond the Centre for Pharmacy Postgraduate Education (CPPE) pathway [40], while others had not and faced challenges when supporting a broad range of patients. This variation in expertise was linked to the autonomy pharmacists felt in pursuing further specialised training in dementia care.

Participants emphasised the value of knowledge sharing and collaborative learning opportunities to enhance clinical pharmacists’ confidence and skills in dementia care. Currently, these resources did not appear to be readily available:

*“It would be good to see what other pharmacists were doing, maybe more specialist dementia pharmacists and see how their role was […]. That would be quite good because it’s nice to see what other pharmacists are doing [...]. I don’t think I have the confidence to do that [put into practice seeing a patient if their disease progressed] yet but, perhaps, after teaching and seeing what other pharmacists were doing, maybe after that, yes.” [CP07]*

Differences in confidence when communicating with people with dementia also influenced care processes. For example, a lack of confidence led to pharmacists only speaking to family carers and not engaging with the person with dementia directly, demonstrating a lack of person-centred care, but also risk of missing important side effects an individual is experiencing:

*“I think because we tend to speak a lot to the carers there tends to be general reluctance to speak directly to dementia patients. I think it might be because we struggle as to how we would deal with that conversation.” [CP13]*

Clinical pharmacists also noted concerns about risks of medication adherence. Many individuals were reported to lack support structures, in particular people with dementia or cognitive impairment living at home without family carers, which could lead to missed doses:

*“I think that’s the struggle with these patients because when they’ve got dementia, either they’re alone and there is no carer or with this patient, he’s [patient with dementia] lucky, he’s got his wife. But they’re also elderly and dealing with their own things.” [CP11].*

Challenges associated with the stigma and acceptance of mental health disorders or cognitive impairment among some patients from ethnic minority backgrounds added further complexity to care processes. This can often result in the risk of not seeking help from services and receiving support from a clinical pharmacist:

*“[I] think with diverse cultures there is a lack of understanding and awareness of mental health disease, but more so with things like dementia, because you’re dealing with cultures who traditionally don’t exist in mental health, you know, mental health doesn’t exist in their cultures, or it’s not spoken about, and there’s a lot of stigma. But then you’re also dealing with the acceptance of the patients that, you know, they’re getting old or they’re having trouble with their memory, so it’s an added layer of complication to get them to understand that.” [CP03]*

Language barriers sometimes further hindered clinical pharmacist’s ability to deliver effective communication and care for patients. Reliance on interpreters was thought to limit the process of developing rapport and having empathetic communication, which may impact delivery of care among minority ethnic groups.

A range of challenges related to the people within the work system, both individual and collective factors, informed care processes and shaped outcomes. Challenges such as poor team cohesion, pharmacists’ confidence and experience, and patient-related factors (stigma, support network, language) interacted to influence the risk of medication-related harm.

### Tools and technology

In the SEIPS 2.0 framework tools and technology are resources used by individuals, such as clinical pharmacists, to support care processes and achieve tasks [33].

Clinical pharmacists reported increasing reliance on digital tools within primary care services, which may disadvantage those unable to access or utilise the internet, such as older people with dementia. This could lead to missed appointments and opportunities for medication reviews. The reliance on these tools risks a complete change in care processes from face-to-face consultation to online, which risk exacerbating inequalities and excluding vulnerable groups such as many of those with dementia:

*“I do think that as General Practice, we need to be really careful about this drive for modern technology. You know, we use “askmyGP,”* [41] *we use all this – we’re talking on Zoom, you know, the virtual aspect. But actually, there’s a cohort of patients out there who that’s a world away from and I think we need to make sure that the good old-fashioned, on the telephone, being seen face-to-face, you know, inviting them into the practice.” [CP01].*

Assessment tools such as the General Practitioner Assessment of Cognition (GPCOG), used by clinical pharmacists and other healthcare professionals alike to screen for cognitive impairment, were reported to be tailored to Western cultures and not always appropriate for patients from minority ethnic backgrounds, leading to potential health inequalities for this group of patients:

*“For example, the GPCOG questionnaire, it asks you to tell something that’s happened in the news in the last week. And for people who are from different ethnic backgrounds, the news they’re watching might not be the news that you’re watching […] just because they haven’t watched BBC News doesn’t mean that they don’t know what’s happening in the world [CP05]*

In summary, ensuring usability and accessibility of digital tools within healthcare is crucial for their effectiveness. Without this, certain tasks, such as accessing information about medications and healthcare, may be unavailable for patients with digital barriers, leading to suboptimal care and increased risk of harm. Furthermore, assessment tools, like the GPCOG, may exhibit cultural bias, potentially leading to inaccurate evaluations and health inequalities for patients from minority ethnic backgrounds.

### Tasks

Tasks refer to the duties undertaken by clinical pharmacists or patients, such as medication reviews or taking prescribed medication. These tasks also have specific attributes, including complexity and variability, which influence the process of conducting medication reviews, for example.

Clinical pharmacists reported encountering substantial challenges that increased task complexity when supporting people with dementia, particularly when deprescribing. GPs were at times said to be reluctant to reduce dosages or discontinue medications, requiring pharmacists to engage in negotiation and persuasion as part of the deprescribing process to justify their recommendations. This was particularly the case for medications for people with dementia with potential adverse effects, such as anti-cholinergic drugs, or when addressing polypharmacy concerns:

*“I’m waiting for the day when they say, drugs with an anticholinergic burden cause dementia, I think we’re quite close to that and can more or less say, you know, don’t give these drugs. But I think that would help me convince more GPs to deprescribe them, if we had that in black and white.” [CP02].*

In addition, despite the potential risks associated with certain medications, some patients were reported to be reluctant to discontinue them, even when deprescribing was recommended. A combination of long-term use and reliance on online information contributed to this:

*“Once they have been on it for years to get someone to stop something, it is really hard. Especially when they are in their 40s and 50s. […] they have got Google as well, they have got all the symptoms so they need to take the drug. It is really hard these days, to get people to stop.” [CP10]*

The shift towards remote appointments introduced further challenges in the medication review process. Participants reported telephone consultations, while overall beneficial, were less effective than face-to-face meetings for comprehensive medication reviews, particularly for people with dementia. They emphasised the importance of home visits for holistic care for those unable to come into a GP clinic, as remote consultations risked missing critical aspects of a patient’s health, such as gait and failing to identify red flags of deterioration:

*“For the vast majority of people, doing a telephone conversation, reviewing their medications, discussing healthy lifestyle, all of that, you can do that on the phone. You can’t read them [person with dementia], the thing that you can’t see is someone walk down a corridor, you can’t see how they sit down and stand up…” [CP06]*

These findings highlight the challenges clinical pharmacists face in the process of performing essential tasks among some patient groups, which can have implications for patient safety. Difficulties in the process of deprescribing may contribute to polypharmacy and medication-related risks when patients do not want change or when GPs are hesitant. The shift to remote consultations limits a pharmacist’s ability to conduct a comprehensive assessment of some patients, increasing the risk of missing signs of harm or deterioration in the person with dementia.

### Organisation

Organisation in the SEIPS 2.0 framework refers to external structures that influence a clinical pharmacist outside of their control, including work schedules, resources, training availability, social norms and organisational culture [33]. Several organisational factors were highlighted which impacted care processes and outcomes for people with dementia and clinical pharmacists.

Variations in training resources and opportunities beyond the CPPE pathway across England contributed to differences in specialist knowledge of long-term conditions, including dementia. These inconsistencies affect the expertise of pharmacists and their ability to provide equitable care, particularly in regions with fewer training opportunities/resources:

*“I wouldn’t have had the training I had unless I was working out of that location; I would never have had that level of training, you know, by attending CPPE. I’ve been on dementia courses before, and this was at another scale.” [CP06]*

Established referral pathways for further support such as health and wellbeing coaches, social prescribers or care navigators, were crucial. However, availability across geographical locations and primary care networks (PCNs) varied and referral pathways were not consistently available across every PCN, impacting care continuity and holistic care for people with dementia:

*“Yes, so we can refer them to a social prescriber, we can refer them to physiotherapists, we can refer – if there’s a need for a secondary referral, then I can bring that up. […] There’s the health and wellbeing coach, as well, so it depends what’s available in our PCN, we would be able to refer them.” [CP07]*

Clear safeguarding pathways were thought essential for clarifying care processes and minimising risk for both people with dementia and pharmacists. Knowledge of these pathways and processes for escalating concerns was important and seemed well-embedded with some pharmacists:

*“Safeguarding patients, if you think that the patient isn’t being supported at home, or there might be issues with a carer, then I think we have a good protocol; I would know what to do if I thought something was wrong, because we have a Safeguarding Lead and we also have adult MDTs once a month, as well, as well as team meetings.” [CP07]*

Moreover, competing demands to conduct medication reviews for people with long-term conditions such as diabetes, asthma, and hypertension were often prioritised over dementia due to limited time, commissioning priorities, and limited dementia care expertise:

*“Because we [pharmacists] do so many different long-term health conditions, I don’t think we can give 100% to dementia, do you know what I mean? For example, if you have a respiratory nurse, all she does is respiratory so she’s an expert in that field, but with pharmacists, I think if there’s something they’re really passionate about, they will be an expert in that field, but mostly, we do everything. And I don’t think dementia has been one of the priorities for quite some time; it’s mostly blood pressure, diabetes and other conditions like asthma and COPD; dementia and heart failure is more specialist.” [CP07]*

Pharmacists reported lacking sufficient time for patient consultations, which limited their ability to conduct comprehensive and safe medication reviews. Short appointments were too brief for many people with dementia, who may have communication difficulties and require additional time, potentially leading to incomplete assessments and missed opportunities for support and intervention:

*“I wish I had more time in the day. Sometimes it’s really frustrating when reception (staff) kind of know that this patient has got memory impairment and they book them in for a standard slot rather than an extended, you know, double slot, that I find really frustrating.” [CP12]*

Regular medication reviews with people with dementia at 3- or 6-month intervals were often hindered because of a lack of capacity among clinical pharmacists, which put patients at risk of suboptimal medication management and increased risk:

*“Infrequent appointments: the change [to medications] is not always getting implemented and being effective three months or six months down the line because you’re not following them up three or six months down the line.” [CP13]*

Clinical pharmacists also raised safety concerns when lone working and completing home visits, particularly when working with patients who are distressed or have behavioural that challenges associated with dementia:

*“I think the other safety consideration is actually going out on these home visits, I quite often go out on my own. […] I had one visit that was particularly scary. I’d actually gone out to visit the husband, who didn’t have dementia, but his wife had dementia. She saw me coming in as a, you know, as a young lady into her house and speaking to her husband, and in her mind she was younger in years, and she saw me as a threat to stealing her husband and she didn’t really want me to leave, you know, that was quite frightening.” [CP12]*

These organisational level challenges highlight potential risks to the patient and pharmacists, because of not having enough time, limited training opportunities or even having to work alone in potentially unsafe situations.

### Internal environment

The characteristics of the environment such as physical layout, noise and available space constitutes the internal environment. There was limited reference to this within the interviews. However, participants highlighted face-to-face consultations in the practice, including home visits, were widely recognised as beneficial in providing holistic and comprehensive medication reviews to ensure patient safety. Space constraints in GP surgeries, meant several clinical pharmacists had to work remotely. This included completing medication reviews and assessments over the phone, which may come with the associated challenges of suboptimal care for people with dementia as important aspects to a person’s care can be missed when assessed remotely:

*“Because a lot of pharmacists don’t always have room in the surgery for them, you know, they’re working remote and even if they are in the surgery sometimes, they haven’t got adequate space.” [CP04]*

### External environment

The wider external environment consisting of macro-level societal and policy factors outside of an organisation can produce processes which can lead to risk for both patients and pharmacists. Health inequalities and poverty impacted patient safety regarding medication adherence. While prescriptions are free in England for people aged 60 years and over or who receive state benefits (and other groups), some patients were unable to afford prescribed medication, particularly for long term conditions requiring ongoing treatment, therefore the process of taking medications was affected for some adults and people with dementia:

*“Universal Credit [state benefit], yes. They can get free prescriptions, which makes life simpler for them. But, some people are on the boundary so they are not getting it [Universal Credit] so prescriptions aren’t free and they are having to pay for them. Then, they have to decide which medication they take.” [CP10]*

Moreover, some clinical pharmacists reported uncertainty about guidelines or protocols specific to support people with dementia. Participants felt unsupported in their role whilst working with people with dementia and mentioned a lack of guidance:

*“I don’t think we have any local guidelines for Dementia. And I think that there isn’t any, apart from NICE (National Institute for Health and Care Excellence) guidelines on what the consultant tells us. There isn’t so much more apart from that and so sometimes I do feel you’re sort of just left alone to look after these patients without some kind of procedure [or] protocol in place.” [CP11]*

For clinical pharmacists supporting residents living in care homes, there were challenges to providing effective care, due to the high turnover of care home staff who sometimes could not give comprehensive handovers to each other. Inconsistencies in information regarding residents sometimes hindered clinical pharmacists’ care management:

*“When I go into the care home […] where they’ve had multiple turnover of staff. The staff might have emailed over the weekend to say, “Oh this thing happened.” You go there on the Monday, nobody knows anything about it.”” [CP12]*

Clinical pharmacists also frequently encountered misunderstandings about their role and services they deliver, with patients often confusing them with community pharmacists:

*“It doesn’t matter if they’ve got dementia or not people just struggle with the role. I’ve been there for a year and a half and there have been other pharmacists there for five years, but people still struggle with the role of there being a pharmacist and what their role because a lot of people think they’re a chemist.” [CP13]*

In summary, there were several societal, regulatory and policy factors outside the organisation which seemed to contribute to patient risk, as identified by clinical pharmacists. They reported a lack of policy and guidelines in dementia care, health inequalities, lack of opportunities to network and lack of understanding of the clinical pharmacy service.

## Discussion

This study reveals that the quality and safety of dementia care delivered by clinical pharmacists in primary care is influenced by a complex interplay of factors spanning individual expertise, team dynamics, access to resources and technology, organisational structures, and the broader health and care environment.

We identified multiple, interconnected key factors within work systems and work processes that contribute to risks in delivering dementia care. These factors included variations in clinical pharmacist training and confidence in their role, language barriers and stigma, a lack of policies, procedures and capacity. These factors shape care processes and can increase risks of harm for both patients and pharmacists, including missed opportunities for identifying decline and ‘red flags’, or safety concerns, as well as possible inappropriate prescribing.

The SEIPS 2.0 framework also highlights an interplay of these factors within work systems; the risks are interconnected, influencing each other in complex ways. For example, a lack of dementia training for clinical pharmacists can lead to geographical variations or a “postcode lottery” in the quality of care (organisation work system), with variations in clinical expertise and confidence across different regions (people work system). Similarly, both individual attributes (pharmacist skills) and available tools and technology (e.g., remote software or digital platforms) can impact how the key tasks, such as medication reviews and deprescribing for people with dementia, are conducted. Furthermore, the consequences of risks can feed back into the work systems, exacerbating the initial problem. For example, suboptimal care for people with dementia, can increase the workload of clinical pharmacists and other primary care professionals, further straining the system. Therefore, risks are not isolated and may create self-perpetuating amplifying challenges and risks across work systems.

Understanding risks and their interactions is crucial for identifying system adaptations that can minimise risk of harm. By understanding the challenges associated with providing dementia care, a proactive approach can be adopted to mitigate further adverse events [42], and healthcare systems can become more adaptive and dynamic [43]. Service delivery by clinical pharmacists can be enhanced and better equipped to reduce the risks of potential harm to people with dementia.

### People

Poor team cohesion and collaboration among healthcare professionals was reported in this research and have historically been well-documented contributors to patient risk and suboptimal care [44,45]. These interprofessional relationships can impact patient experiences, for example as shown in this study leading to suboptimal prescribing caused by challenges in deprescribing, such as uncertainty of where responsibility lies [46]. Open and collaborative discussions and sharing values around deprescribing can improve working relationships and can optimise safe medication use by patients [47].

Communication barriers were noted to extend to patients whose first language was not English. Communication and meeting patient needs effectively are important prerequisites for successful patient outcomes and healthcare access [48]. Language and cultural barriers were reported to contribute to miscommunication, including the ability to perform cognitive assessments adequately, decreasing the quality of care delivered and patient safety [49].

Insufficient training, confidence and variations in expertise and skills were identified as barriers for clinical pharmacists in their provision for people with dementia. Similar barriers for health care professionals that increase patient risk are reported in other research, including lack of communication skills [50,51], lack of awareness of patient-carer relationships [52] and insufficient training on multiple long-term conditions [50], such as dementia [53]. Learning from others was recommended by several participants to enhance confidence and skills in dementia care. Interprofessional training and collaboration were also recommended by the Commission on Education and Training for Patient Safety (NHS) to enhance patient safety [54].

Our results align with previous studies in observing that certain health conditions, such as dementia, may carry stigma within minority ethnic communities that prevent seeking support from primary care teams [55,56]. These patient groups are therefore at heightened risk of poor medication adherence and lack of support from clinical pharmacists. We have shaped the below discussion according to the key domains within the work system of SEIPS 2.0.

### Tools and technology

Tools and technology were discussed less in the interviews, however participants did note a growing reliance on online services poses significant barriers to older people, especially those with dementia, as approximately one in four older people lack internet access [57]. Relying on online platforms can exacerbate health inequalities by excluding individuals without digital literacy or resources. Future research may wish to consider tools and technology in more detail, to explore this in more depth.

### Tasks

Clinical pharmacists in our research reported encountering challenges when attempting a variety of tasks including discontinuing medications that were potentially harmful, as well as general medication management for people living with dementia and older people. These challenges related to collaboration with other healthcare staff, e.g. GPs, as well as some patients’ reluctance to discontinue long-term medications. Older people in particular have been reported to express reluctance to discontinue medications, even when these medications pose significant risks [58,59]. Their doctor’s influence and experience in deprescribing can significantly impact the success of medication interventions [60]. There is also evidence of delays in implementing medication changes recommended by dementia specialists highlighting further systemic issues within healthcare systems [61].

Remote appointments pose challenges for holistic care, particularly for people with dementia. Evidence also suggests limited opportunities for the task of comprehensive assessment during remote appointments and an increased risk of safeguarding concerns about vulnerable patients [62]. This thereby exacerbates health inequalities and compromises the inclusion of the voice of the person with dementia [63,64]. While carers can support communication during a consultation, it is important to acknowledge the complexities and potential challenges associated of relying solely on carers. Families may have differing perspectives influenced by culture or personal beliefs [48] which could compromise patient autonomy or result in carers not acting in the individual’s best interests. Additionally, relying solely on carers may lead to missed identification of important medications side-effects that the individual has not reported or displayed [63].

### Organisation

This study highlights competing demands faced by clinical pharmacists to support people with other long-term health conditions for pharmacists to address in consultations, that means dementia lacks priority. Other research has highlighted systemic and structural barriers facing patients accessing post-diagnostic dementia care, such as a lack of clear pathways and limited knowledge of local services and professional roles [65] and competing health care priorities that take precedence over dementia [25]. While there are policies to support dementia care and guidelines for healthcare professionals, including clinical pharmacists, many gaps and inequalities persist [66,67]. The observation in our research of a lack of local guidelines or protocols specifically for managing dementia care in the clinical pharmacy role highlights a potential gap. While general guidelines and protocols for older people’s healthcare exist, they may not be consistently applied by clinical pharmacists to address the specific needs of people with dementia. This “othering” of people with dementia could be attributed to a lack of awareness or training on how to effectively apply existing general guidelines to the specific needs of people with dementia.

### Internal and external environment

Moreover, health inequalities affecting those from deprived backgrounds bring challenges and risks associated with unsustainable medication adherence as highlighted in our research. Despite the NHS in England providing exemption from prescription charges to people who meet specific criteria [68], notably all those aged 60 + , some patients below that age do not meet these criteria and consequently, there is a risk of medication non-adherence due to it being unaffordable [69].

Time and capacity limitations were identified as barriers for clinical pharmacists to provide care to the wide range of patients they support, but particularly for people with dementia who may need longer time with a clinician. Capacity limitations have been demonstrated to be a barrier to patient safety in other research and can lead to less detailed explanations to patients, potentially leaving patients feeling ignored and with unanswered questions [70,71].

### Implications for policy and practice

The wide-ranging sources of patient risk of harm indicates complex and multifaceted initiatives must be utilised to improve patient care and safety. The importance of team cohesion, confidence, autonomy and the availability of standardised training were all important for clinical pharmacists to be able to work to their best ability and support people with dementia. Training that aims to increase confidence and communication skills when conversing with people with dementia should be paramount. It was suggested by our participants that there is benefit from interpersonal learning, and this type of training should be available across England to foster collaboration and professional development.

### Strengths and limitations

This is the first study to review clinical pharmacists’ experience of risks associated with providing dementia care in England. The SEIPS 2.0 framework is useful in providing a view of the whole healthcare system in relation to patient risk. Alternative frameworks may have been useful, such as The Human Factors Analysis and Classification System (HFACS) [72], however the SEIPS framework offers a granular view and is well embedded within NHS practice [73]. It offers a framework to explore the different aspects of a work system and their interactions that contribute to patient risk, rather than focusing on only one aspect of the work system and treating it in isolation [74].

However, our study is limited in the conclusion which can be applied to other healthcare systems internationally as the sample was drawn exclusively from England, so the findings may not be representative of risks facing people with dementia within different healthcare systems. Our sample was also small-sized and did not explore the perspectives of other parties such as General Practitioners, other members of the primary care team, patients and carers.

## Conclusion

We have highlighted the complex and interconnected risks within work systems, which influence dementia care, using a systems engineering and safety approach. A proactive management strategy is encouraged to mitigate these risks, adapt service delivery, and enhance patient safety and the effectiveness of clinical pharmacist care for people with dementia.

## Supporting information

S1 File DCPharm interview guide pharmacist. (DOCX)

## References

[1] Vincent C, Amalberti R. Safety Strategies in Primary Care. [online]. Springer; 2016. Available from: https://www.ncbi.nlm.nih.gov/books/NBK481870/.

[2] Sideman AB, Alagappan C, Hernandez de Jesus A, Ma M, Dohan D, Rosen HJ, et al. Challenges and approaches when addressing dementia in the context of chronic comorbid conditions in primary care settings. 2023.

[3] El-Saifi N, Moyle W, Jones C, Tuffaha H. Medication adherence in older patients with dementia: a systematic literature review. J Pharm Pract. 2018;31(3):322–34. doi: 10.1177/0897190017710524 28539102

[4] Black BS, Johnston D, Leoutsakos J, Reuland M, Kelly J, Amjad H, et al. Unmet needs in community-living persons with dementia are common, often non-medical and related to patient and caregiver characteristics. Int Psychogeriatr. 2019;31(11):1643–54. doi: 10.1017/S1041610218002296 30714564 PMC6679825

[5] Fox C, Maidment I, Moniz-Cook E, White J, Thyrian JR, Young J, et al. Optimising primary care for people with dementia. Ment Health Fam Med. 2013;10(3):143–51. 24427181 PMC3822661

[6] NT Contributor. Achieving person-centred dementia care through a bio-psychosocial model | Nursing Times. [online] Nursing Times. 2020. [cited 28 Oct. 2024]. Available from: https://www.nursingtimes.net/dementia/achieving-person-centred-dementia-care-through-a-bio-psychosocial-model-08-12-2020/

[7] Keast S, Broatch JR, Chung S, Dixon R, Dongol R, Emerson L, et al. Best practice in dementia health care: Key clinical practice pointers from a national conference and innovative opportunities for pharmacy practice. Res Social Adm Pharm. 2024;20(10):1014–21. doi: 10.1016/j.sapharm.2024.07.005 39122588

[8] Härlein J, Dassen T, Halfens RJG, Heinze C. Fall risk factors in older people with dementia or cognitive impairment: a systematic review. J Adv Nurs. 2009;65(5):922–33. doi: 10.1111/j.1365-2648.2008.04950.x 19291191

[9] Gould L, Tilly J, Reed P. Dementia care practice recommendations for professionals working in a home setting: phase 4. Chicago, IL: Alzheimer’s Association Publication; 2009.

[10] Delgado J, Jones L, Bradley MC, Allan LM, Ballard C, Clare L, et al. Potentially inappropriate prescribing in dementia, multi-morbidity and incidence of adverse health outcomes. Age Ageing. 2021;50(2):457–64. doi: 10.1093/ageing/afaa147 32946561

[11] Dickins M, Goeman D, O’Keefe F, Iliffe S, Pond D. Understanding the conceptualisation of risk in the context of community dementia care. Soc Sci Med. 2018;208:72–9. doi: 10.1016/j.socscimed.2018.05.018 29772396

[12] Ayre MJ, Lewis PJ, Keers RN. Understanding the medication safety challenges for patients with mental illness in primary care: a scoping review. BMC Psychiatry. 2023;23(1). doi: 10.1186/s12888-023-04850-5 PMC10258931 37308835

[13] Onder G, Lattanzio F, Battaglia M, Cerullo F, Sportiello R, Bernabei R, et al. The risk of adverse drug reactions in older patients: beyond drug metabolism. Curr Drug Metab. 2011;12(7):647–51. doi: 10.2174/138920011796504563 21495971

[14] Marengoni A, Onder G. Guidelines, polypharmacy, and drug–drug interactions in patients with multimorbidity. BMJ. 2015;350:h1059. doi: 10.1136/bmj.h1059 25761379

[15] Growdon ME, Gan S, Yaffe K, Steinman MA. Polypharmacy among older adults with dementia compared with those without dementia in the United States. J Am Geriatr Soc. 2021;69(9):2464–75. doi: 10.1111/jgs.17291 34101822 PMC8440349

[16] Fralick M, Bartsch E, Ritchie CS, Sacks CA. Estimating the use of potentially inappropriate medications among older adults in the United States. J Am Geriatr Soc. 2020;68(12):2927–30. doi: 10.1111/jgs.16779 32841366

[17] NHS England (n.d.). NHS England» Clinical Pharmacists. [online]. Available from: https://www.england.nhs.uk/gp/expanding-our-workforce/cp-gp/

[18] Oboh L, Leon C, Qadir S, Smith F, Francis S-A. Frail older people with multi-morbidities in primary care: a new integrated care clinical pharmacy service. Int J Clin Pharm. 2018;40(1):41–7. doi: 10.1007/s11096-017-0566-8 29222733

[19] Birt L, Dalgarno L, Poland F, Wright D, Bond C. What happens when pharmacist independent prescribers lead on medicine management in older people’s care homes: a qualitative study. BMJ Open. 2023;13(10):e068678. doi: 10.1136/bmjopen-2022-068678 37907299 PMC10619113

[20] Anderson M, Francetic I. Adoption of clinical pharmacist roles in primary care: longitudinal evidence from English general practice. Br J Gen Pract. 2025;75(752):e173–80. doi: 10.3399/BJGP.2024.0320 39317390 PMC11800411

[21] Wang T, Benedict N, Olsen KM, Luan R, Zhu X, Zhou N, et al. Effect of critical care pharmacist’s intervention on medication errors: a systematic review and meta-analysis of observational studies. J Crit Care. 2015;30(5):1101–6. doi: 10.1016/j.jcrc.2015.06.018 26260916

[22] Manias E, Williams A, Liew D. Interventions to reduce medication errors in adult intensive care: a systematic review. Br J Clin Pharmacol. 2012;74(3):411–23. doi: 10.1111/j.1365-2125.2012.04220.x 22348303 PMC3477343

[23] MacDonald S, Grand JH, Caspar S, Macdonald SW. Clinical features and multidisciplinary approaches to dementia care. J Multidiscip Healthc. 2011;4:125–47. doi: 10.2147/JMDH.S17773 21655340 PMC3104685

[24] Ye HMY, Xiao LD, Ullah S, Chang RHC. Hospital nurses perceived challenges and opportunities in the care of people with dementia: a mixed-methods systematic review. J Clin Nurs. 2024;3(8):2849–84. doi: 10.1111/ajag.12827 38544319

[25] Bernstein Sideman A, Al-Rousan T, Tsoy E, Piña Escudero SD, Pintado-Caipa M, Kanjanapong S, et al. Facilitators and barriers to dementia assessment and diagnosis: perspectives from dementia experts within a global health context. Front Neurol. 2022;13:769360. doi: 10.3389/fneur.2022.769360 35418934 PMC8997042

[26] Komwong D, Greenfield G, Hayhoe B. Clinical pharmacists in primary care: a safe solution to the workforce crisis? J Royal S Med. 2018;111(4). 10.1177/0141076818756618 PMC5900835 29480743

[27] McDerby N, Kosari S, Bail K, Shield A, Peterson G, Naunton M. Pharmacist-led medication reviews in aged care residents with dementia: a systematic review. Australas J Ageing. 2020;39(4):e478–89. doi: 10.1111/ajag.12827 32748980

[28] xxx.

[29] Iflaifel M, Lim RH, Ryan K, Crowley C. Resilient Health Care: a systematic review of conceptualisations, study methods and factors that develop resilience. BMC Health Serv Res. 2020;20(1):324. doi: 10.1186/s12913-020-05208-3 32303209 PMC7165381

[30] Burnand A, Woodward A, Kolodin V, Manthorpe J, Jani Y, Orlu M, et al. Service delivery and the role of clinical pharmacists in UK primary care for older people, including people with dementia: a scoping review. BMC Prim Care. 2025;26(1):10. doi: 10.1186/s12875-024-02685-x 39810119 PMC11731431

[31] Agostini L, Onofrio R, Piccolo C, Stefanini A. A management perspective on resilience in healthcare: a framework and avenues for future research. BMC Health Serv Res. 2023;23(1):774. doi: 10.1186/s12913-023-09701-3 37468875 PMC10357696

[32] NHS England. Patient Safety. [cited 2025 Jan 14]. Available from: https://www.england.nhs.uk/patient-safety/

[33] Holden RJ, Carayon P, Gurses AP, Hoonakker P, Hundt AS, Ozok AA, et al. SEIPS 2.0: a human factors framework for studying and improving the work of healthcare professionals and patients. Ergonomics. 2013;56(11):1669–86. doi: 10.1080/00140139.2013.838643 24088063 PMC3835697

[34] Werner NE, Rutkowski R, Graske A, Finta MK, Sellers CR, Seshadri S, et al. Exploring SEIPS 2.0 as a model for analyzing care transitions across work systems. Appl Ergon. 2020;88:103141. doi: 10.1016/j.apergo.2020.103141 32421635 PMC7400988

[35] Martinez VI, Marquard JL, Saver B, Garber L, Preusse P. Consumer health informatics interventions must support user workflows, be easy-to-use, and improve cognition: applying the SEIPS 2.0 model to evaluate patients’ and clinicians’ experiences with the CONDUIT-HID intervention. Int J Hum–Comput Interact. 2017;33(4):333–43. doi: 10.1080/10447318.2016.1278340

[36] Marcilly R, Forrierre J, Thébault J, Bayen S, Grimes T. System engineering approach of the home self-management of type 2 diabetes: a work in progress. In: Proceedings of the European Conference on Cognitive Ergonomics 2024. 2024. pp. 1–5. doi: 10.1145/3673805.3673840

[37] Braun V, Clarke V. Thematic analysis: A practical guide: Sage; 2021.

[38] Malterud K, Siersma VD, Guassora AD. Sample size in qualitative interview studies: guided by information power. Qual Health Res. 2016;26(13):1753–60. doi: 10.1177/1049732315617444 26613970

[39] Lumivero. NVivo (Version 14). 2023. Available from: www.lumivero.com

[40] Clinical pharmacists in general practice education: introduction to phases. [online]. 2019. Available from: https://www.cppe.ac.uk/career/clinical-pharmacists-in-general-practice-education#navTop

[41] askmyGP. askmyGP | How it works - for the patients, and for the practice. [online] Available from: https://askmygp.uk/how-it-works/

[42] Sampson P, Back J, Drage S. Systems-based models for investigating patient safety incidents. BJA Educ. 2021;21(8):307–13. doi: 10.1016/j.bjae.2021.03.004 34306732 PMC8283417

[43] Anderson JE, Ross AJ, Back J, Duncan M, Snell P, Hopper A, et al. Beyond “find and fix”: improving quality and safety through resilient healthcare systems. Int J Qual Health Care. 2020;32(3):204–11. doi: 10.1093/intqhc/mzaa007 32108882

[44] Dickins M, Goeman D, O’Keefe F, Iliffe S, Pond D. Understanding the conceptualisation of risk in the context of community dementia care. Soc Sci Med. 2018;208:72–9. doi: 10.1016/j.socscimed.2018.05.018 29772396

[45] Keast S, Broatch JR, Chung S, Dixon R, Dongol R, Emerson L, et al. Best practice in dementia health care: key clinical practice pointers from a national conference and innovative opportunities for pharmacy practice. Res Social Adm Pharm. 2024;20(10):1014–21. doi: 10.1016/j.sapharm.2024.07.005 39122588

[46] Brodaty H, Green A. Who cares for the carer? The often forgotten patient. Aust Fam Physician. 2002;31(9):833–6. 12402702

[47] Parslow RM, Duncan LJ, Caddick B, Chew-Graham CA, Turner K, Payne RA, et al. Collaborative discussions between GPs and pharmacists to optimise patient medication: a qualitative study within a UK primary care clinical trial. Br J Gen Pract. 2024;74(748):e727–34. doi: 10.3399/BJGP.2024.0190 38950941 PMC11466292

[48] Hadziabdic E, Heikkilä K, Albin B, Hjelm K. Problems and consequences in the use of professional interpreters: qualitative analysis of incidents from primary healthcare. Nurs Inq. 2011;18(3):253–61. doi: 10.1111/j.1440-1800.2011.00542.x 21790876

[49] Al Shamsi H, Almutairi AG, Al Mashrafi S, Al Kalbani T. Implications of language barriers for healthcare: a systematic review. Oman Med J. 2020;35(2):e122. doi: 10.5001/omj.2020.40 32411417 PMC7201401

[50] Campbell P, Torrens C, Pollock A, et al. A scoping review of evidence relating to communication failures that lead to patient harm. 2018. Available from: https://www.gmc-uk.org/-/media/documents/a-scoping-review-of-evidence-relating-to-communication-failures-that-lead-to-patient-harm_p-80569509.pdf

[51] Shitu Z, Hassan I, Thwe Aung M, Tuan Kamaruzaman T, Musa R. Avoiding medication errors through effective communication in a healthcare environment. Malays J Mov Health Exerc. 2018;7(1):115. doi: 10.4103/2600-9404.323043

[52] Longtin Y, Sax H, Leape LL, Sheridan SE, Donaldson L, Pittet D. Patient participation: current knowledge and applicability to patient safety. Mayo Clin Proc. 2010;85(1):53–62. doi: 10.4065/mcp.2009.0248 20042562 PMC2800278

[53] Surr CA, Gates C, Irving D, Oyebode J, Smith SJ, Parveen S, et al. Effective dementia education and training for the health and social care workforce: a systematic review of the literature. Rev Educ Res. 2017;87(5):966–1002. doi: 10.3102/0034654317723305 28989194 PMC5613811

[54] Health Education England. Improving safety through education and training. [online] Health Education England. 2016. Available from: https://www.hee.nhs.uk/sites/default/files/documents/Improving%20safety%20through%20education%20and%20training.pdf

[55] Ye S, Redwood S, et al. Crazy person is crazy person. It doesn’t differentiate: an exploration into Somali views of mental health and access to healthcare in an established UK Somali community. Int J Equity Health. 2020;19(1):190. 33109227 10.1186/s12939-020-01295-0 PMC7592587

[56] Nielsen TR, Nielsen DS, Waldemar G. Barriers in access to dementia care in minority ethnic groups in Denmark: a qualitative study. Aging Ment Health. 2021;25(8):1424–32. doi: 10.1080/13607863.2020.1787336 32619352

[57] New research from Age UK reveals almost 6m older people can’t access the internet safely - Rural Services Network. [online]. [cited 2023 Oct 20]. Available from: https://www.rsnonline.org.uk/new-research-from-age-uk-reveals-almost-6m-older-people-cant-access-the-internet-safely

[58] Weir KR, Shang J, Choi J, Rana R, Vordenberg SE. Factors important to older adults who disagree with a deprescribing recommendation. JAMA Netw Open. 2023;6(10):e2337281. doi: 10.1001/jamanetworkopen.2023.37281 37819657 PMC10568363

[59] Weir KR, Naganathan V, Carter SM, Tam CWM, McCaffery K, Bonner C, et al. The role of older patients’ goals in GP decision-making about medicines: a qualitative study. BMC Fam Pract. 2021;22(1):13. doi: 10.1186/s12875-020-01347-y 33419389 PMC7796626

[60] Linsky A, Simon SR, Stolzmann K, Meterko M. Patient attitudes and experiences that predict medication discontinuation in the Veterans Health Administration. J Am Pharm Assoc (2003). 2018;58(1):13–20. doi: 10.1016/j.japh.2017.10.012 29154017 PMC6788281

[61] Maidment ID, Damery S, Campbell N, Seare N, Fox C, Iliffe S, et al. Medication review plus person-centred care: a feasibility study of a pharmacy-health psychology dual intervention to improve care for people living with dementia. BMC Psychiatry. 2018;18(1):340. doi: 10.1186/s12888-018-1907-4 30340480 PMC6194710

[62] Rosen R, Wieringa S, Greenhalgh T, Leone C, Rybczynska-Bunt S, Hughes G, et al. Clinical risk in remote consultations in general practice: findings from in-COVID-19 pandemic qualitative research. BJGP Open. 2022;6(3). doi: 10.3399/BJGPO.2021.0204 35487581 PMC9680756

[63] Tuijt R, Rait G, Frost R, Wilcock J, Manthorpe J, Walters K. Remote primary care consultations for people living with dementia during the COVID-19 pandemic: experiences of people living with dementia and their carers. Br J Gen Pract. 2021;71(709):e574–82. doi: 10.3399/BJGP.2020.1094 33630749 PMC8136581

[64] Neves AL, Li E, Gupta PP, Fontana G, Darzi A. Virtual primary care in high-income countries during the COVID-19 pandemic: policy responses and lessons for the future. Eur J Gen Pract. 2021;27(1):241–7. doi: 10.1080/13814788.2021.1965120 34431426 PMC8404680

[65] Wheatley A, Bamford C, Brunskill G, Booi L, Dening KH, Robinson L. Implementing post-diagnostic support for people living with dementia in England: a qualitative study of barriers and strategies used to address these in practice. Age Ageing. 2021;50(6):2230–7. doi: 10.1093/ageing/afab114 34240114 PMC8675435

[66] Alzheimer’s Disease International. World Alzheimer Report 2016. Improving Healthcare for People with Dementia. London: Alzheimer’s Disease International; 2016.

[67] Alzheimer’s Disease International. World Alzheimer Report 2019: Attitudes to Dementia. London: Alzheimer’s Disease International; 2019.

[68] National Health Service. NHS prescription charges. [online]. 2024 Available from: https://www.nhs.uk/nhs-services/prescriptions/nhs-prescription-charges/

[69] Morgan SG, Lee A. Cost-related non-adherence to prescribed medicines among older adults: a cross-sectional analysis of a survey in 11 developed countries. BMJ Open. 2017;7(1):e014287. doi: 10.1136/bmjopen-2016-014287 28143838 PMC5293866

[70] Schildmeijer K, Nilsen P, Ericsson C, Broström A, Skagerström J. Determinants of patient participation for safer care: a qualitative study of physicians’ experiences and perceptions. Health Sci Rep. 2018;1(10). doi: 10.1002/hsr2.87 PMC6266354 30623042

[71] Sarkhosh S, Abdi Z, Ravaghi H. Engaging patients in patient safety: a qualitative study examining healthcare managers and providers’ perspectives. BMC Nurs. 2022;21(1):374. doi: 10.1186/s12912-022-01152-1 36581873 PMC9801597

[72] Shappell SA, Wiegmann DA. The Human Factors Analysis and Classification System--HFACS. Scholarly Commons. 2018. Available from: https://commons.erau.edu/publication/737/11718505

[73] NHS England. Patient Safety Incident Response Framework supporting guidance Guide to responding proportionately to patient safety incidents [online]. 2022. Available from: https://www.england.nhs.uk/wp-content/uploads/2022/08/B1465-3.-Guide-to-responding-proportionately-to-patient-safety-incidents-v1-FINAL.pdf

[74] Carayon P, Schoofs Hundt A, Karsh BT, Gurses AP, Alvarado CJ, Smith M, et al. Work system design for patient safety: the SEIPS model. Quality and Safety in Health Care. 2006;15(suppl_1):i50–8. 10.1136/qshc.2005.015842 PMC2464868 17142610

