# Supplementary material for: Identifying risks in dementia care: Insights from a qualitative study with clinical pharmacists
Source: PLoS One. 2025 Jun 24;20(6):e0325472. doi: 10.1371/journal.pone.0325472 (PMC12186929; doi:10.1371/journal.pone.0325472)
Supplement: S1 File — (DOCX) [file pone.0325472.s001.docx]

**DCPharm Topic Guide: Clinical Pharmacists**

**Introduction**

- Thank participant for taking part, introduce self.
- Briefly explain purpose of research: To explore the role of clinical pharmacists in primary care and dementia care so that we can understand the future potential of clinical pharmacists working with people with dementia and their carers.
- Recap consent form (if completed separate to interview):
- Participation is voluntary – can stop interview at any time or choose not to answer questions.
- Withdrawing data – up to six weeks after interview
- Confidentiality – unless risk of harm to you or others.
- Anonymity – personal details not shared, not identifiable in reports/publications.
- Recording – for quotes, and to check accuracy.
- Length of interview: up to 60 minutes

**Background**

1. **What are your main roles and responsibilities as a clinical pharmacist?**
   1. What types of cases/patients do you see? (i.e. local demographic)
   2. What services do you currently provide?
   3. Medicine reconciliation, risk management - Probe on:

- prescription review (i.e. appropriateness of prescribing, overprescribing/deprescribing),

- medication use reviews (appropriateness of the medicines for the patient e.g. assessing formulation, adherence)

- patient counselling

- prescribing themselves (as non-medical/ independent prescribers in specific clinics or repeat prescriptions)

- 1. In what ways do you work with other health care professionals/community pharmacists/pharmacy technicians? (e.g. involved in joint consultation with a geriatrician or neurologist?)

1. **What opportunities do you have in your current role for providing support to people, including those living with dementia?**
   1. New services?
   2. General support/support specific to dementia?
   3. What would you change about how you currently work or do things?

**Support for people with dementia**

1. **Could you tell me about a recent patient you saw who had dementia or memory problems and how you were involved in their care?**
   1. At what points were you involved in their care e.g., diagnosis/post diagnosis, end of life?
   2. Probe specific examples of care and support e.g.

- support patient with medicine taking e.g. formulations to aid swallowing or use of medicine
- supporting carer e.g. aide memoirs/ reminder charts/ medicines compliance aids

1. **What specific support and care do you think a clinical pharmacist can offer a person with dementia (and their family)?**
   1. Annual dementia reviews?
   2. What do you think some of the benefits are of including a pharmacist in the care of patients with dementia?
   3. What would you like to offer that you can’t currently (e.g. are there missed opportunities?)
   4. How might the care and support you provide for people living with dementia be improved?

**Challenges in providing support**

1. **Can you tell me about some of the challenges that clinical pharmacists face when providing support and care for people living with dementia?** (either from personal experience or other known issues) Probe examples if needed:
   1. Time constraints/capacity in role
   2. Communication – pwd unable to express themselves, challenges of communicating with a dyad or multiple family members? Assessing capacity and understanding needs?
   3. Communication with patients including those with diverse ethnic background (BAME)?
   4. Tailoring needs/approaches for different cultures/culturally sensitive or relevant support and care.
   5. Clinical complexity – linked to multi-morbidity (f), unstable nature of dementia, or difficulty of prognosis and diagnosis.
   6. Multi-morbidity and polypharmacy
   7. Working with other clinicians. Awareness of role among other professional groups.
   8. Training (appropriate for needs/fit for purpose?)
   9. Young onset dementia? How does this differ?
2. **What do you think the most significant risks are in relation to supporting and providing care for someone with dementia?** (*if not mentioned, probe on patient safety, clinical practice risk, risk to professional and clinical practice)*
   1. How do you try to manage and minimise these risks? (is this always possible?)
   2. Do you have protocols and procedures in place? If so, what has informed these e.g., guidelines, guidance notes, local practice/pcn resources?
   3. Supporting diverse groups i.e., culturally sensitive or relevant care?
3. **Can you tell me about any training you have accessed relating to dementia and/or older adult care?** (draw upon responses to Q5 if any challenges raised around training)
4. **Can you tell me about anything that would help you further in providing the care and support needed for people living with dementia?** e.g.,
   1. Further understanding of disease progression and symptoms, hands on experience, an experienced colleague to guide you, formal training, understanding of formulations (e.g. if patient has swallowing difficulties).
   2. Any interventions to protect patient autonomy or supporting family carers?
   3. Practical support such as decision aids? Communication tools? What would they look like or include?
   4. Greater cultural understanding of different ethnic communities?
5. **When supporting fellow clinical pharmacists, what key piece of advice or support would you give about providing care and support for people living with dementia and family carers?**
6. **How would you tell a person living with dementia about the role of a clinical pharmacist?** (probe for example of a time when they have had to do this)

End of interview follow up questions

- Do you feel you could cope with the length of the interview?
- Did you find talking in the interview helpful?
- Did you feel the interview caused you distress?
- Did you find talking in the interview helpful?
